# Supplementary material for: Graphene Quantum Dots as Intracellular Imaging-Based Temperature Sensors
Source: Materials (Basel). 2021 Jan 29;14(3):616. doi: 10.3390/ma14030616 (PMC7866248; doi:10.3390/ma14030616)
Supplement: Supplementary file 1 [file materials-14-00616-s001.pdf]

materials

MDPI

Supplementary Materials

Graphene Quantum Dots as Intracellular Imaging-Based Temperature Sensors

Bong Han Lee [1], Ryan Lee McKinney [1], Md. Tanvir Hasan [1,2] and Anton V. Naumov [1,*]

[1] Department of Physics and Astronomy, Texas Christian University, Fort Worth, TX 76129, USA; bong.lee@tcu.edu (B.H.L.); ryan.l.mckinney@tcu.edu (R.L.M.); tanvir.hasan@tcu.edu (M.T.H.)

[2] Current affiliation: Biosystems and Biomaterials Division, National Institute of Standards and Technology, Gaithersburg, MD 20899, USA

* Correspondence: a.naumov@tcu.edu

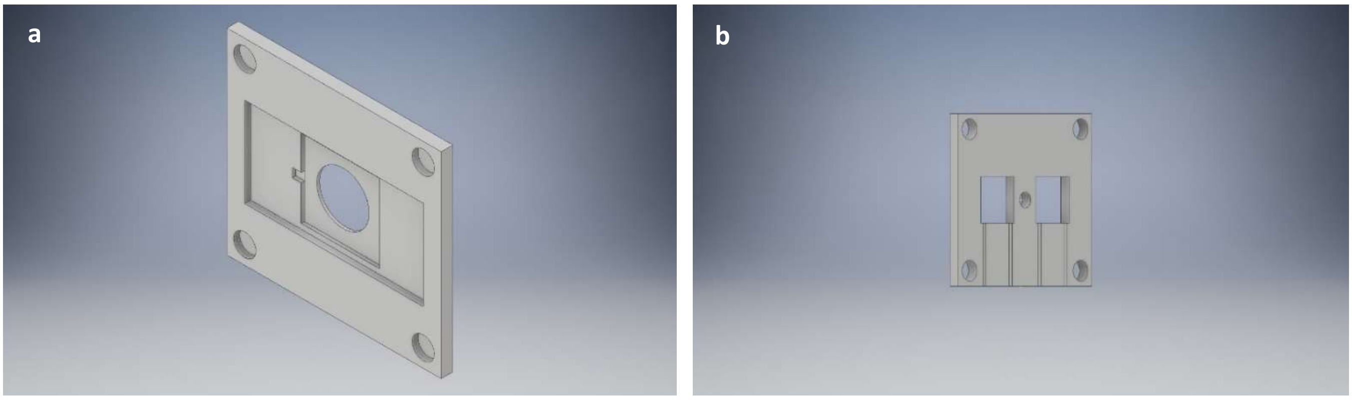

Figure S1. Schematic of the ITMD for temperature-controlled microscopy imaging. **(a)** Base of the holder intended for cover slips, copper plate for improved heat conductivity and a thermocouple for in situ temperature measurements **(b)** top of thermal device that holds Peltier thermoelectric modules. (Autodesk Pro.).

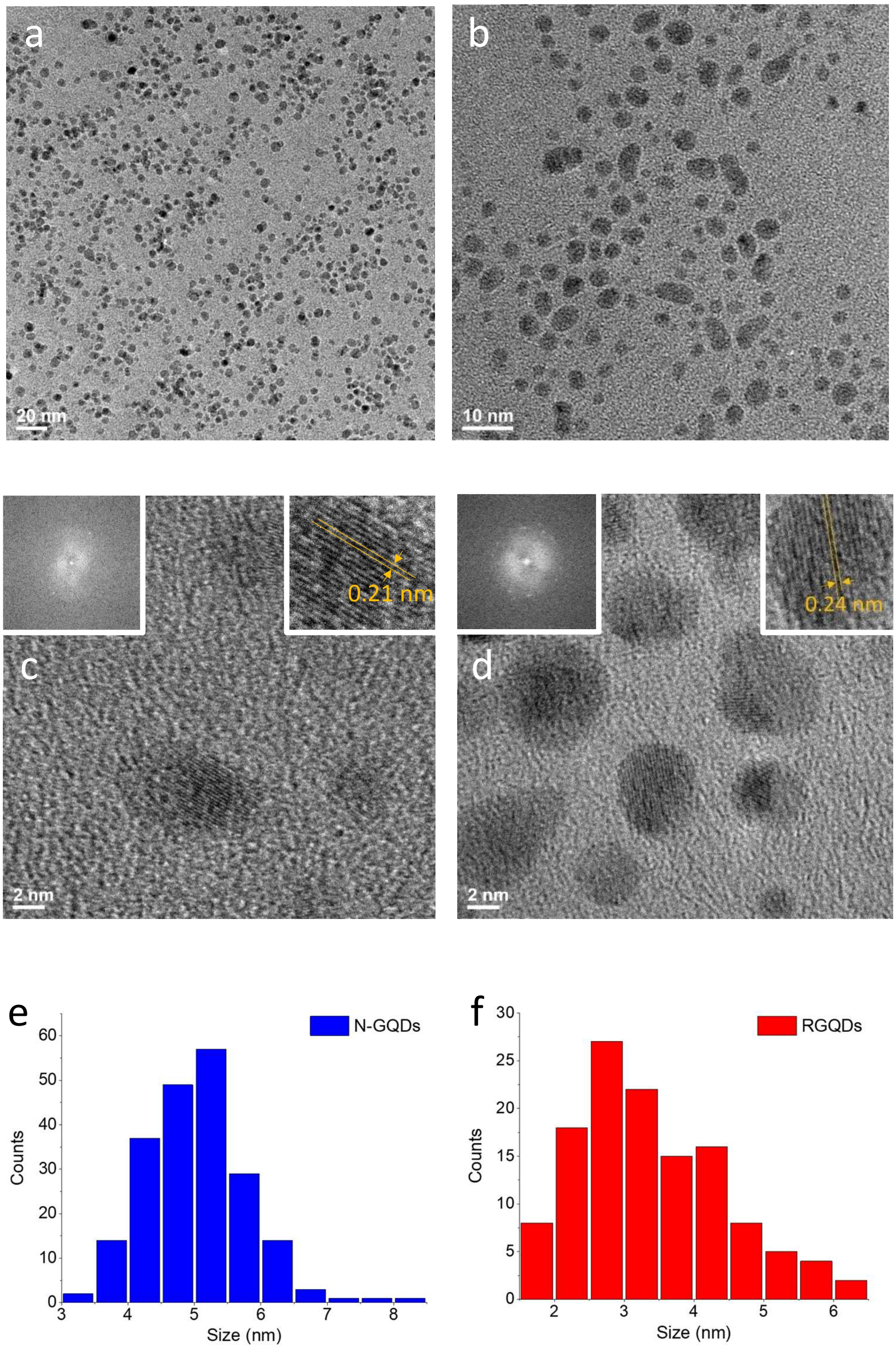

Figure S2. TEM images of as-synthesized N-GQDs **(a)** and RGQDs **(b)**. HRTEM images of as-synthesized N-GQDs **(c)** and RGQDs **(d)** with corresponding insets showing diffraction pattern with discernable lattice fringes (indicating crystallinity) and zoom-in on an individual GQD showing the lattice spacing corresponding to graphene lattice. Size distribution of N-GQDs **(e)** and RGQDs **(f)** with a size of 5.0 ± 0.7 and 3.4 ± 1.1 nm (mean ± s.d.), respectively.

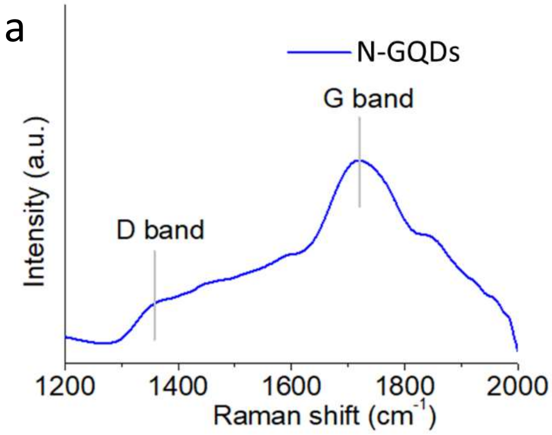

Figure S3. Raman spectra of N-GQDs **(a)** and RGQDs **(b)**.

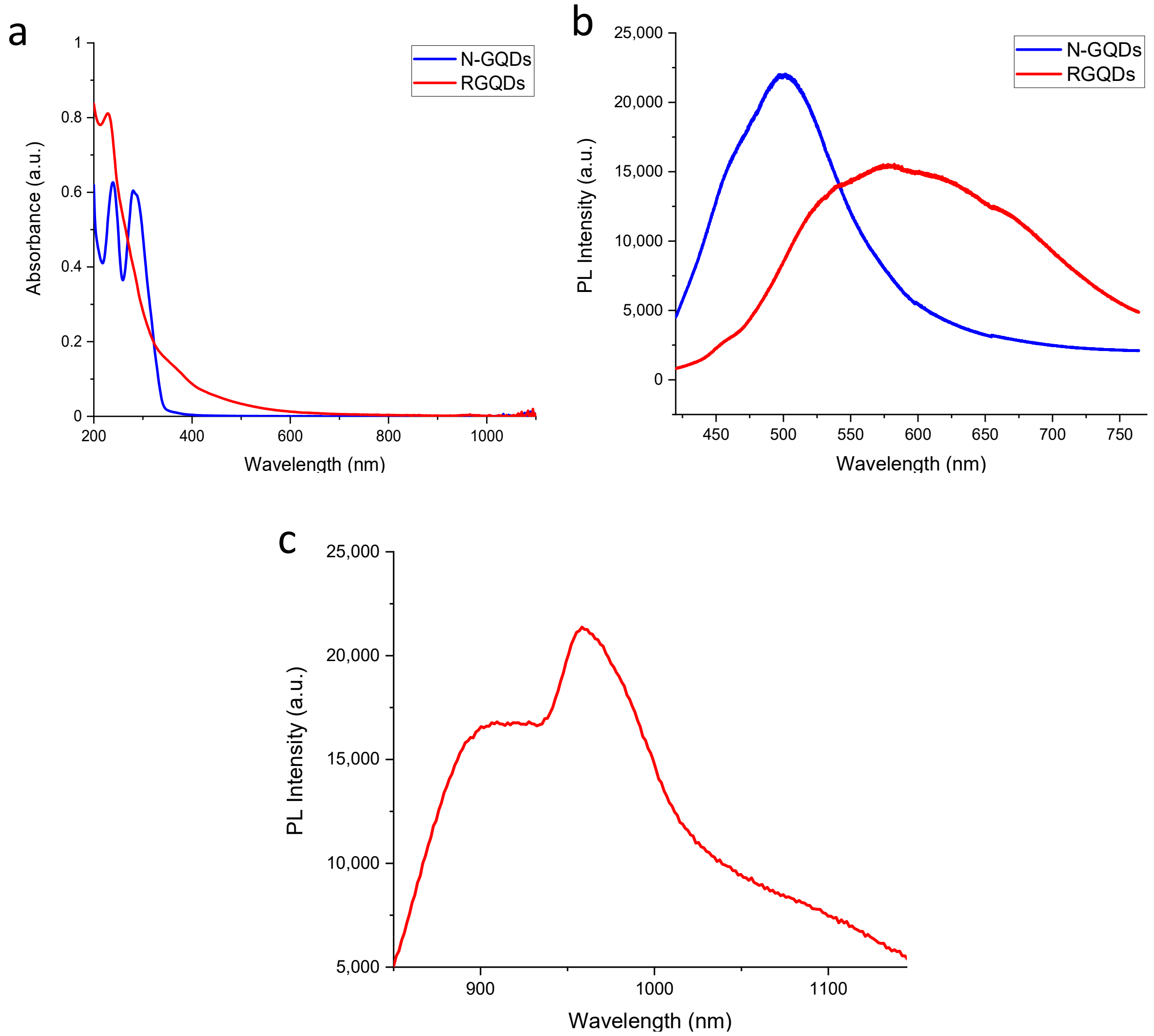

Figure S4. **(a)** Absorption of N-GQDs (blue) and RGQDs (red). **(b)** Visible fluorescence emission of N-GQDs (blue) and RGQDs (red) with 400 nm excitation. **(c)** NIR fluorescence emission of RGQDs with 475 nm excitation.

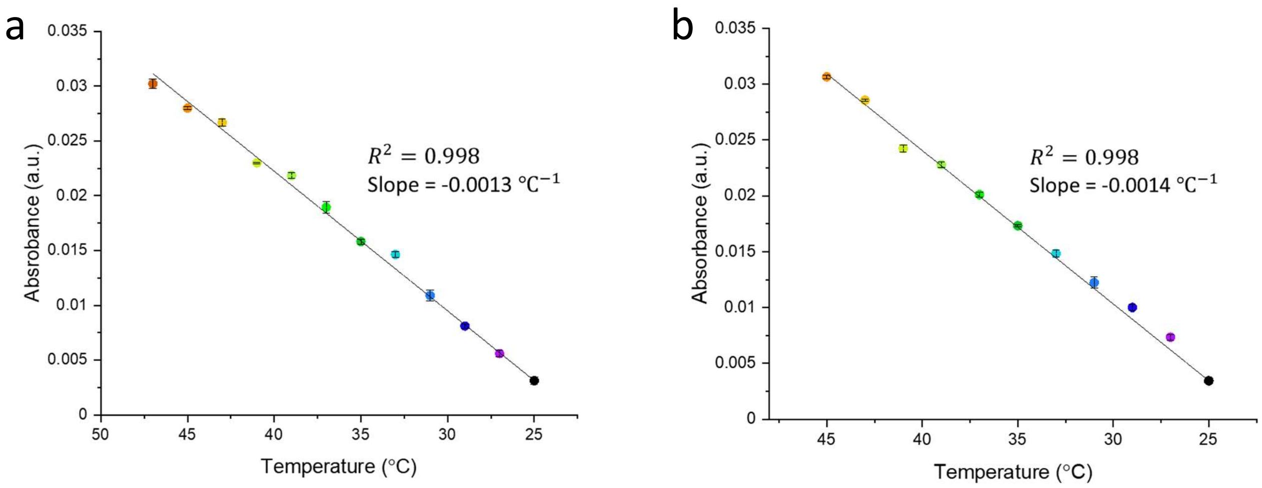

Figure S5. Near-infrared absorbance peak value vs. temperature for the cooling process for **(a)** N-GQDs and **(b)** RGQDs.

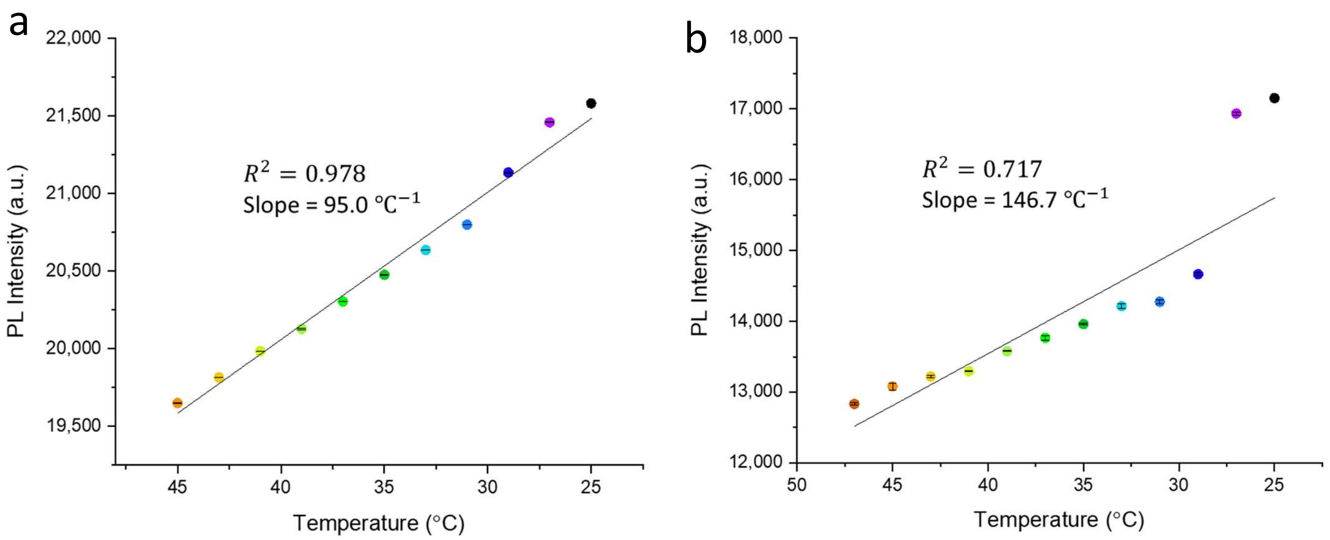

Figure S6. Peak photoluminescence intensity vs. temperature for the cooling process of RGQDs NIR **(a)** and visible **(b)** fluorescence.

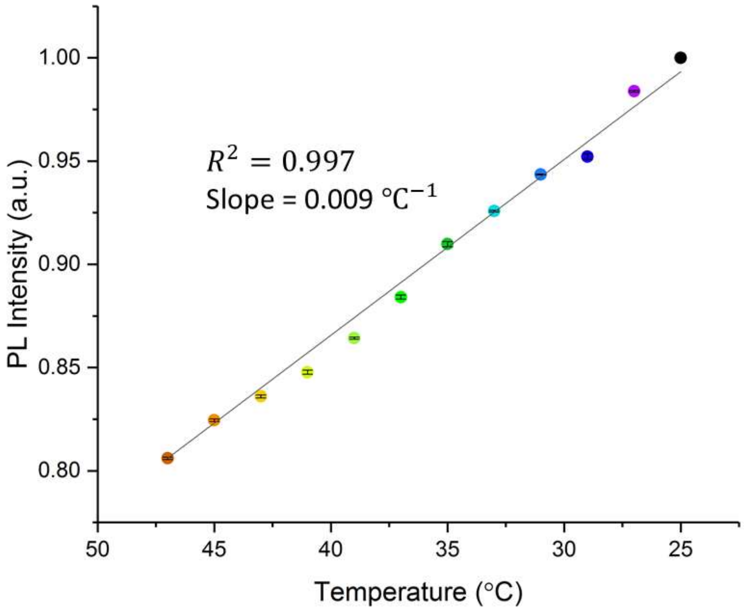

Figure S7. Visible peak photoluminescence intensity vs. temperature for the cooling process of N-GQDs.

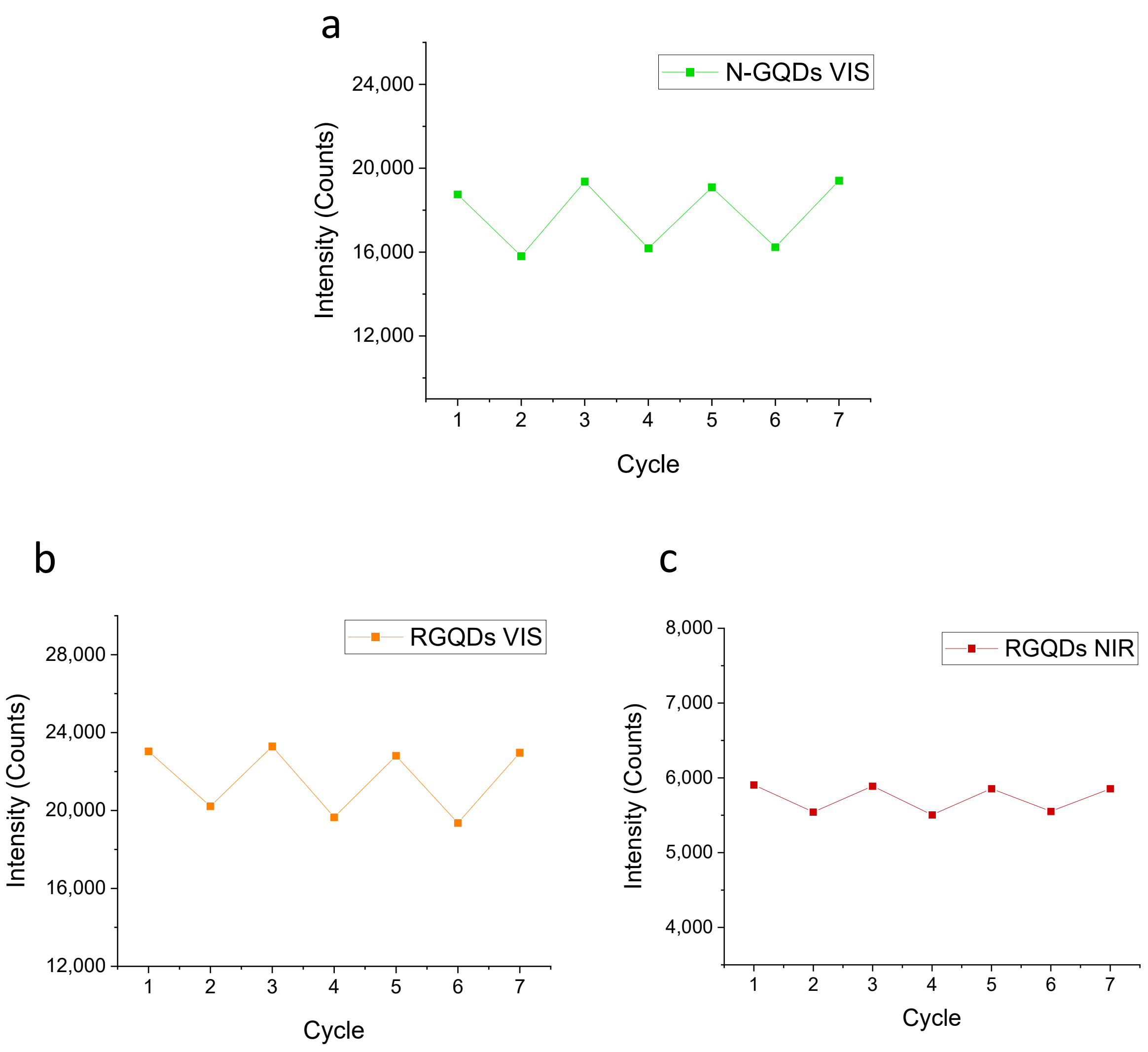

Figure S8. Heating and cooling cycles of the visible N-GQDD fluorescence (**a**) visible RGQD fluorescence (**b**) and NIR RGQD fluorescence (**c**) between 25 °C and 49 °C.
